# Knowledge, attitude and practices towards cervical cancer control among University students in Kilimanjaro region, Tanzania

**DOI:** 10.1371/journal.pone.0327879

**Published:** 2025-07-11

**Authors:** Alex Mremi, Aron P. Joseph, Donatila V. Njunwa, Doris A. Ntupwa, Doris G. Rwenyagila, Joseph Mlay

**Affiliations:** 1 Department of Pathology, Kilimanjaro Christian Medical Centre, Moshi, Tanzania; 2 School of Medicine, KCMC University, Moshi, Tanzania; 3 Kilimanjaro Clinical Research Institute, Moshi, Tanzania; 4 Department of Obstetrics and Gynecology, Kilimanjaro Christian Medical Centre, Moshi, Tanzania; University of Environment and Sustainable Development, GHANA

## Abstract

**Background:**

Cervical cancer (CC) is a major public health issue in the sub-Saharan Africa region, partly due to limited public awareness among women at risk. University students are part of the age group that is at risk. Assessing their knowledge, attitude, and practices (KAP) about CC, its causes, and prevention methods can reveal gaps that need to be addressed through education. We aimed to assess KAP towards CC control and their determinants among University students in Tanzania.

**Methods:**

A cross-sectional survey was conducted among 708 undergraduate students in three Universities, using a pre-tested questionnaire. Descriptive statistics and binary logistic regression were used to describe variables and identify associations between variables; a p-value of <0.5 determined the association.

**Results:**

The majority of students (75.7%) were not knowledgeable, and 82.5% had a negative attitudes regarding CC. Only 25.4% had ever been screened against CC. Being female and a medical student in ≥4^th^ year of studying were significantly associated with good knowledge and attitude of CC (p < 0.000), while other socio-demographic characteristics didn’t show any association.

**Conclusion:**

The findings indicate that Tanzanian University students’ lack the basic knowledge and attitudes towards CC prevention and control, highlighting the need for comprehensive strategies to ensure no woman is left behind.

## Introduction

Cancer of the cervix (CC) is a global public health issue. It is the most fatal form of cancer in developing nations, the majority of which are in sub-Saharan Africa (SSA) region [1]. In the year 2020, about 604,000 new CC cases were diagnosed worldwide, and 342,000 CC-related deaths were reported, out of which SSA is the leading [2]. Tanzania ranked fourth in the world for having the highest rate of CC in 2020, with an estimated over 60 new cases per 100,000 women [3]. The number of deaths from CC was also high in that year, at the rate of about 42 per 100,000 women. The high prevalence of human immunodeficiency virus/acquired immunodeficiency syndrome (HIV/AIDS) in Tanzania is thought to be a contributing factor to the high incidence of CC [3].

The human papillomavirus (HPV) is a necessary but not sufficient causative agent for CC. HPV is primarily transmitted through sexual intercourse. Two HPV types (types 16 and 18) are believed to contribute to up to 70% of all CC cases [4]. In addition, epidemiological studies have consistently demonstrated that smoking, early sexual debut, prolonged use of oral contraceptives, low socioeconomic status, multi-parity, and risky sexual behaviors increase the risk of CC [5]. Clinically, CC disease is characterized by abnormal vaginal bleeding during sexual intercourse or between menstrual periods, pelvic pain, and foul-smelling vaginal discharge. The Tanzanian government, through the Ministry of Health, in line with the World Health Organization (WHO), has put in place several intervention strategies to attain the 5-year goal (2020–2024) of the national CC prevention and control program [6].

Undergraduate University students undergo a critical transition into adulthood, shaping independent lifestyle choices, including health-seeking behavior such as cancer screening, which can persist into adulthood and impact long-term health and non-communicable disease risk [7]. The uptake of CC is influenced by the individual’s educational level and socio-demographic characteristics [8]. Individuals, particularly those with University education, are believed to have favorable knowledge, attitudes, and the best practices towards CC control [8]. While University students often fall into the age group that is at high risk for HPV infection, a previous study [9] has indicated many University students do not have adequate knowledge about CC, its risk factors, and the importance of early detection. This can lead to a lack of participation in preventive measures, such as HPV vaccination and regular CC screening, resulting in neglecting these critical health practices [10]. Without proper education, students may not fully understand the importance of safe sexual practices and HPV vaccination. In some cultures, CC and HPV-related topics may be stigmatized, leading to further misinformation and reluctance to seek preventive care [10]. Addressing these attitudes is crucial for effective public health interventions. Similarly, a positive attitude towards preventive healthcare can significantly reduce the incidence of CC. However, misconceptions about or a lack of awareness of CC may negatively influence people, causing them to seek treatment from inappropriate sources, including traditional healers, which may increase the delay in appropriate care for CC and thus leads to poor treatment outcomes [11–13].

SSA countries face different problems such as illiteracy challenges, poor socio-economics, and even poor policy, all of which make it difficult for the nations to fight against CC [8]. Having adequate and the right knowledge about CC is significantly associated with high screening rates among females as an early way of preventing and controlling CC [10]. Also, knowledge about CC influences individual perceptions about the disease, for instance, perceptions about benefits, susceptibility, severity or seriousness, and barriers [11]. Additionally, beliefs and attitudes are potential proxies for knowledge and therefore predict screening practices for CC control. Negative beliefs and attitudes towards CC reduce the likelihood of CC screening uptake. Due to poor beliefs and misinformation, most women in developing countries tend to seek treatment from traditional healers, which in turn increases the delay in appropriate care and thus leads to poor treatment outcomes.

Universities are ideal settings for health education initiatives. Addressing knowledge gaps and promoting positive attitudes towards CC prevention can have a lasting impact on students’ health behaviors. A number of studies in SSA have assessed the level of knowledge, attitude, and practice (KAP) towards CC prevention and control in females of reproductive age groups, and these studies’ results indicated very low KAP levels [8–10,13,14]. But there is a dearth of studies regarding the KAP of CC in risk groups such as the high-learning student population, despite the growing burden of CC in Tanzania. Data from such studies are essential for planning CC control measures. Thus, we conceived this cross-sectional survey to determine the level of KAP towards CC control and their determinants among University students in Tanzania.

## Materials and methods

### Study design and time

A cross-sectional survey was performed in three Universities (one medical and the other two non-medical) in Kilimanjaro, Tanzania, from June 2022 to March 2024. The Kilimanjaro region is one of Tanzania’s 31 administrative regions. According to the 2022 national census, the population of the Kilimanjaro region was estimated to be 1,917,302 [15]. There are many Universities and colleges that serve a large number of people both within and outside the region, including international students.

### Study population

Until early this year, 2024, the Tanzania Commission for Universities (TCU) has accredited and thus registered about 43 Universities [16]. Of these, five are in the Kilimanjaro region [17]. Three out of five Universities in the Kilimanjaro region, namely KCMC University, Moshi Co-operative University (MoCU), and Mwenge Catholic University (MWECAU) were randomly chosen for this research. These Universities were chosen because they provide a target population of interest for this study. This implies that the three institutions are typical or illustrative of the population under investigation. In addition, these institutions are logistically accessible in terms of location, resources, and the current research team members’ presence. Similarly, the institutions provide support for the research through permissions, infrastructure, and collaboration. MoCu is among the oldest Universities in Tanzania. It is a public institution that was founded in 1963. Primarily, it is responsible for training human resources in cooperatives, community development, and business studies at the certificate, diploma, degree, master’s degree, and PhD levels. On the other hand, KCMC University is one of the major private medical Universities in Tanzania, having been founded in 1997. MWECAU is also a private University, offering various education programs at various levels [17]. Students in these institutions are basically from all parts of the country. Both sexes (female and male) of undergraduate students aged ≥18 years who were voluntarily willing to participate in the study were involved. Male students were assessed on knowledge and attitude towards CC only, while female students were assessed the same as males but with an additional section of screening practices against CC prevention. The study excluded postgraduate students as well as students under the age of 18 years.

### Sample size estimation

Sample size estimation was derived from a population with a single proportion at a 95% confidence level (CI), Z (1-ά/^2^) = 1.96, a 30% expectation for the proportion of individuals with suboptimal knowledge, and a 5% margin of error based on data from previous studies. Following the assumption above, the sample size was calculated as follows:


$$\text{N}=\frac{{\text{Z}}_{1-\alpha /2}\cdot \text{P}(1-\text{P})}{{\text{L}}^{2}}$$

Where:

N = Minimum sample size required

Z _1-α/2_ = Standard normal value corresponding to 95% confidence interval Z = 1.96

P = the estimated prevalence of participants was assumed to be 30% based on other studies.

L = Margin of error 5% (0.05)

$\text{n}=\frac{{\left(1.96\right)}^{2}\left(0.30\mathrm{\backslash rightleft}(0.70\right)}{0.05^{2}}$

N = 322

In order to have a minimal sample size of females needed for a separate analysis of CC screening and practices, the sample size was doubled, i.e., n = 644. Adding 15% of non-response to the minimum sample size gives us a sample size of 740.

### Sampling technique

The study involved a multistage sampling technique as previously described [18–22]. In the first stage, the Universities in the Kilimanjaro region were broadly grouped into health versus non-health as well as public versus private categories. In the second stage, a representative sample of Universities was purposively selected within each category. Within each selected University, a random selection of degree programs was done. Within each University, the study participants were selected from each year and program course using a systematic random sampling technique for every 10^th^ student from each school’s registrar office logbook until the required sample size was obtained. From each University, an equal number of students were recruited so as to ensure that each subgroup is fairly represented in the study, ensuring statistical comparisons are more valid and straightforward, since unequal samples may skew results and complicate the use of statistical tests. Random sampling with equal sample sizes can reduce sampling bias, especially in stratified random sampling. It ensures that no group dominates the data due to its size in the population. Equal sample sizes are critical for minimizing confounding variables and ensuring that any observed effect is not due to sample size differences. Three faculties, namely the faculty of Medicine, faculty of Nursing, and faculty of Rehabilitation, were selected out of five at KCMC University, while at MoCU, three faculties, namely Business and Information Science, Community Development, and Cooperative, were selected out of 10. Similarly, three faculties from MWECAU, namely the Faculties of social science, education, and arts, were selected out of 15. Considerations were also made for the students’ programs and the duration of their studies.

### Data collection methods, tools, and study procedure

A self-administered online questionnaire was employed to gather various socio-demographic information as well as KAP towards CC control. The questionnaire was designed using previous related research [8–11,14]. The questionnaire was developed in English and translated into Kiswahili (the national language). In the present study, Cronbach’s alpha coefficient for the knowledge and attitude questionnaire was 0.84, suggesting acceptable internal consistency [18].

### Operational definitions

Knowledge about CC was tested by 14 questions on general knowledge and perception of risk factors, symptoms, and the screening process. Attitude was evaluated with 13 questions, of which 9 were on a Likert scale from 1 to 5 points with choices of “strongly disagree, disagree, not sure, agree, and strongly agree.” Four questions, each worth a maximum of four points, were used to assess the screening history, practices, procedures, frequency, and intention to screen for CC if given an opportunity [14]. Only female participants were permitted to respond to this section of the survey. The socio-demographic characteristics, including age, sex, University, year of study, course/program, and marital status, served as independent variables. The KAP scores in relation to CC prevention and control served as dependent variables. The cut-off points for good knowledge, attitude, and screening practice independently were the means of the score so as to reflect the average performance [22]. Participants whose scores were below the mean were regarded as having poor KAP, while those with a mean score equal to the mean value or above were categorized as having good KAP, respectively.

### Data procedures

The investigating team, which included authors in this study, explained to the participants the study’s objectives. Prior to recruitment, study subjects had to sign an informed consent form. On a regular basis, the data questionnaires were assessed for completeness by dedicated data clerks. Attempts were made to trace participants who provided incomplete information in the questionnaires through their study IDs.

### Data analysis plan

STATA software version 17 was used to analyze the data. Tables were used to present the results. Confounding and effect modification were evaluated by observing the regression coefficient variation greater than or equal to 15%, and multicollinearity was checked using the variance inflation factor with a value of *<* 10 as a cut point. All variables that showed statistically significant results with KAP for CC in bivariate logistic regression were entered into multivariate logistic regression to identify the independent contribution of each explanatory variable. The variables that were entered into the final model were primarily identified based on the study objectives, data quality, statistical significance, collinearity, theory and prior knowledge, practical considerations, and domain expertise. Binary logistic regression was used to identify associated factors with our three outcome variables (KAP) regarding CC, its risk factors, and prevention. To illustrate how strongly the result and the explanatory variables are related, the odds ratio at 95% confidence intervals (CI) was computed [14]. A p-value of *<*0.05 was considered a statistically significant association between the independent and dependent variables.

### Ethics statement

The study protocol was reviewed and approved by the research ethics committee of KCMC University (Ethical Approval Number 2466). Confidentiality was maintained throughout the study by using anonymous study IDs instead of names; thus, the data collection tools and the data analysis didn’t include any identification of participants. The participants were aware of their right to voluntarily participate in or withdraw from the study at any stage throughout the study.

## Results

### Socio-demographic characteristics of respondents

Of the 740 participants targeted in this study, only 708 provided complete data in the study, making the response rate 95.7%. Thus, 708 participants were included in this study. Their ages ranged from 18 to 40 years, with a mean age of 22.9 ± SD = 2.4. The distribution of participants by year of study was as follows: 25.1% of them were in their first year of studies, while 42.4% were in their second year, 30.2% in their third year, and 2.3% were in their ≥fourth year, respectively. Detailed baseline information is displayed in Table 1.

**Table 1:** Baseline characteristics of study participants (N = 708).

Variable	n	%
**Age (Mean ± SD)**	(22.9 ± 2.4)	
18-25	652	92.1
26-33	48	6.8
34-41	8	1.1
**Sex**		
Male	354	50.0
Female	354	50.0
**University**		
Medical university (KCMC University)	244	34.5
Non-medical university (MoCU and MWECAU)	464	65.5
**Year of study**		
1	178	25.1
2	300	42.4
3	214	30.2
≥ 4	16	2.3
**Marital status**		
Married	22	3.1
Not married (single)	642	90.7
Cohabiting	44	6.2

### Knowledge of cervical cancer among study participants

With regards to overall knowledge assessment about CC, the vast majority (86.7%) of respondents agreed that they have heard about CC from various sources of information. About 37% of the participants received information regarding CC from health institutions. Slightly over half (51.7%) didn’t know that sexually transmitted viruses (HPV) are the causative agents of CC. More than three-quarters (79.9%) knew about risk factors for CC, and about two-thirds (69.5%) knew at least one or more signs and symptoms of CC. Only 67% knew that CC can be cured at an earlier stage. Nearly two-thirds (62.7%) were not knowledgeable about the presence of the national CC screening program; only 70.1% knew the eligible age for CC screening. Only 16.1% of the respondents had overall good knowledge of CC, i.e., had scored equal to or above the mean value. The vast majority (83.9%) had poor knowledge (Table 2).

**Table 2:** Assessment of University students’ knowledge of cervical cancer, (N = 708).

Variable	Category	n	%
Have you ever heard about CC?	Yes	614	86.7
	No	94	23.3
Where did you learn about CC?	Health institutions	260	36.7
	News media	152	21.5
	Teachers	106	15.0
	Family, friends, neighbors	98	13.8
	Magazines	26	3.7
	I don’t know	66	9.3
What cases CC?	HPV-virus	342	48.3
	Bacterial infection	80	11.3
	Fungus	22	6.2
	Parasites	22	3.1
	I don’t know	220	31.1
Do you know the risk factor(s) for CC?	Yes (mentioned one or more factor)	566	79.9
	I don’t know	142	20.1
What is/are the symptom(s) of CC?	Mentioned one or more sign	492	69.5
	I don’t know	216	30.5
How can someone prevent herself against developing CC?	Mentioned one or more preventive measures	580	81.9
	I don’t know	128	18.1
Which age group (years) in Tanzania has the highest risk of developing CC?	≤19	96	13.6
	20 - 29	252	35.6
	30 - 49	184	26.0
	50 - 69	50	7.1
	≥70	8	1.1
	I don’t know	118	16.7
Which screening method(s) do you know?	Mentioned one or more method	366	51.7
	I don’t know	342	48.3
Do you know if Tanzania has a national program for CC screening?	I know	264	37.3
	I don’t know	444	62.7
If so, what age are Tanzanian women supposed to be screened for CC?	30-50 years	504	71.2
	≥50 years	204	28.8
Are you aware of the national vaccination program to prevent CC?	Yes	278	39.3
	No	430	60.7
If yes, at what age is this offered?	≤8 years 0r ≥ 15 years	393	55.5
	9-14 years	315	44.5
Can CC be successfully treated when detected in its early form?	It can	474	66.9
	It cant	36	5.1
	I don’t know	198	28.0
What are treatment approaches for CC?	Mentioned one or more methods	508	71.8
	I don’t know	200	28.2
Compute knowledge score on CC	Good	114	16.1
	Poor	594	83.9

Keyword: CC- Cervical cancer.

### Attitude of University students on cancer of the cervix

The majority of the respondents (75.7%) had an overall poor perception towards CC and its seriousness. For instance, 70.3% did not think that screening helps in the prevention of CC. Similarly, the vast majority of the female respondents (82.5%) perceived that they were not at risk of getting CC. If given the opportunity to be screened, a considerable proportion were either not sure (14.7%) or not ready (6.2%), respectively (Table 3).

**Table 3:** Attitude of University students toward cancer of the cervix (N = 708).

Variable	Category	n	%
Do you think you are can recognize symptoms of CC?	Strongly uncertain	80	11.3
	Not very certain	268	37.9
	Fairly certain	256	36.2
	Very certain	104	14.7
Do you believe that early detection of CC is beneficial?	Strongly disagree	230	32.5
	Disagree	60	8.5
	Not sure	120	16.9
	Agree	78	11.0
	Strongly agree	220	31.1
Do you believe CC is the major cause of cancer death?	Strongly disagree	196	27.7
	Disagree	100	14.1
	Not sure	240	33.9
	Agree	86	12.1
	Strongly agree	86	12.1
Do you believe that CC can be treated?	Strongly disagree	258	36.4
	Disagree	86	12.1
	Not sure	176	24.9
	Agree	78	11.0
	Strongly agree	110	15.5
Do you believe that screening aids in CC prevention and control?	Strongly disagree	230	32.5
	Disagree	86	12.1
	Not sure	182	25.7
	Agree	64	9.6
	Strongly agree	142	20.1
Do you think there’s chance you could develop CC? (n = 354)	Strongly disagree	94	26.6
	Disagree	46	13.0
	Not sure	152	42.9
	Agree	34	9.6
	Strongly agree	28	7.9
Do you think only women with risky sexual behavior should be regularly screened for CC?	30-50	496	70.1
	>50	88	12.4
	No idea	124	17.5
If you get a chance to screen for cervical cancer, would you be ready for it? (n = 354)	No	22	6.2
	Yes	280	79.1
	I don’t know	52	14.7
Overall positive attitude towards Cervical Cancer	Favorable	24.3	172
	Unfavorable	75.7	536

### Relationship between University students’ knowledge of cancer of the cervix and socio-demographic features

Socio-demographic characteristics were evaluated to see if they correlated with CC knowledge. Marital status, sex, and participants’ age were not significantly associated with CC knowledge. However, being in a medical University and studying in the ≥ 4^th^ year were 60.6 and 26.7 times more likely to be associated with good knowledge of CC as compared to the other counterpart groups, and the difference was statistically significant (95%; CI = 18.4–199.9, and 4.7–152.3), respectively (Table 4).

**Table 4:** Bivariable regression output table on knowledge of cervical cancer among University students (N = 708).

Variable	Total	Knowledge		AOR (95% CI)	P-value
		Good (%)	Poor (%)		
**Age**					
18-25	652	98 (15.0)	554 (85.0)	1.0	
26-33	48	12 (25.0)	36 (75.0)	1.9 (0.7-4.9)	0.202
34-41	8	4 (50.0)	4 (50.0)	5.7 (0.8-41.1)	0.087
**Sex**					
Male	354	60 (16.9)	294 (83.1)	1.1 (0.6-2.00)	0.665
Female	354	54 (15.3)	300 (87.7)	1.0	
**University**					
Medical university*	244	108 (44.3)	136 (55.7)	60.6 (18.4-199.9)	0.000
Non-medical university	464	6 (1.3)	458 (98.7)	1.0	
**Year of study**					
Year 1	178	18 (10.1)	160 (89.9)	1.0	
Year 2	300	38 (12.7)	262 (87.3)	1.3 (0.6-3.0)	0.554
Year 3	214	46 (21.5)	168 (78.5)	2.4 (1.1-5.6)	0.066
Year 4 and above*	16	12 (75.0)	4 (25.0)	26.7(4.7-152.3)	0.000
**Marital status**					
Not married (single)	642	102 (15.9)	540 (84.1)	1.0	
Married	22	4 (18.2)	18 (81.8)	1.176 (0.3-5.6)	0.838
Cohabiting	44	8 (18.2)	36 (81.8)	1.176 (0.4-3.6)	0.777

*significantly associated factors.

### Association between demographic characteristics and attitudes toward cervical cancer assessment

Students’ attitudes towards the CC assessment did not show any association with their age, University, year of study, or marital status. But female students were more likely than their male student counterparts to have a positive attitude (95%; CI 0.2 (0.1–0.4); p < 0.000), Table 5.

**Table 5:** Bivariable regression output table on attitude towards cervical cancer among University students (N = 708).

Variable	Total	Attitude		AOR (95% CI)	p-value
		Positive (%)	Negative (%)		
**Age**					
18-25	652	272 (41.7)	380 (58.3)	1.0	
26-33	48	14 (29.2)	34 (70.8)	0.6 (0.2-1.4)	0.232
34-41	8	4 (50.0)	4 (50.0)	1.4 (0.2-10.0)	0.740
**Sex**					
Female*	354	84 (23.7)	270 (76.3)	0.2 (0.1-0.4)	0.000
Male	354	206 (58.2)	148 (41.8)	1.0	
**University**					
Medical university*	244	120 (49.2)	124 (50.8)	1.7 (1.1-2.6)	0.023
Non-medical university	464	170 (36.6)	294 (63.4)	1.0	
**Year of study**					
Year 1	178	84 (47.2)	94 (52.8)	1.0	
Year 2	300	126 (42.0)	174 (58.0)	0.8 (0.5-1.4)	0.435
Year 3	214	76 (35.5)	138 (64.5)	0.6 (0.4-1.1)	0.099
Year 4 and above	16	4 (25.0)	12 (75.0)	0.4 (0.1-1.9)	0.242
**Marital status**					
Single	642	270 (42.1)	372 (57.9)	1.0	
Married	22	6 (27.3)	16 (72.7)	0.5 (0.1-1.9)	0.336
Cohabiting	44	14 (31.8)	30 (68.2)	0.6 (0.3-1.6)	0.349

*significantly associated factors.

### Screening practices against cervical cancer among University students

Screening practices against CC were assessed among female respondents, of whom only 90 (25.4%) out of 354 respondents had ever been screened for CC. Of these, only 38 (42.2%) of them had a regular screening frequency. Socio-demographic characteristics, which include age, University, year of study, and marital status, didn’t show any significant association with screening practices for CC prevention (Table 6).

**Table 6:** Relationship between social-demographic traits and cervical cancer screening practices among female University students (N = 354).

Variable	Total	Screening practices		AOR (95% CI)	p-value
		Good (%)	Poor (%)		
**Age**					
18-25	336	54 (16.1)	282 (83.9)	1.0	
26-33	16	4 (25.0)	12 (75.0)	1.7 (0.3-9.1)	0.511
34-41	2	0 (0.0)	2 (100.0)	0.0 (0.0)	1.00
**University**					
Medical university	138	24 (17.4)	114 (82.6)	1.0	
Non-medical university	216	34 (15.7)	182 (84.3)	0.9 (0.4-1.9)	0.772
**Year of study**					
Year 1	80	14 (17.5)	66 (82.5)	1.0	
Year 2	164	22 (13.4)	142 (86.6)	0.7 (0.3-2.1)	0.551
Year 3	100	20 (20.0)	80 (80.0)	1.2 (0.4-3.4)	0.763
Year 4 and above	10	2 (20.0)	8 (80.0)	1.2 (0.1-12.2)	0.890
**Marital status**					
Not married (single)	328	56 (17.1)	272 (82.9)	1.0	
Married	12	2 (16.7)	10 (83.3)	0.9 (0.1-8.6)	0.979
Cohabiting	14	0 (0.0)	14 (100.0)	0.00 (0.00)	0.999

## Discussion

Basic knowledge of CC screening is key to reducing deaths secondary to CC. In this study, the vast majority, 614 (86.7%), of respondents agreed that they have heard about CC from different information sources. Previous studies in Africa and Asia have reported lower CC awareness rates. For instance, Maitanmi et al. (2023), in Nigeria, reported that the majority of the respondents (68.4%) had a substantial awareness and knowledge of CC screening [8]. Usman et al. (2023), in Uganda, highlighted that only 56.5% of female University students had knowledge of CC screening [10]. In Saudi Arabia, Abdel-Aziz et al. (2024) demonstrated that 88.7% of respondents had unsatisfactory knowledge about CC [11]. In their study, Manikandhan et al. (2019) established that 69.39% of the professional female Indian college students were not aware of CC [14]. Tadesse et al. (2022), in Ethiopia, highlighted that only 14.8% of the respondents had overall good knowledge of CC screening [18]. Meanwhile, Mruts et al. (2018) observed that only 40.5% of the study participants had heard of CC, but only 35.6% had good knowledge of CC [20], whereas Aweke et al. (2017) reported that nearly half of the respondents (46.3%) had poor comprehensive knowledge of CC [22]. Ruddies et al. (2020), in their study that was conducted in Ethiopia, documented that only 36% of respondents were aware of CC and 4.7% knew symptoms [23]. None of the women named HPV as a risk factor. In a South African study, Hoque et al. (2008) reported that only 6% of the respondents knew the risk factors of CC, whereas less than half (49%) of them knew that a Pap smear is used for the prevention of CC [24]. The differences amongst these studies and our study may be accounted for by variations in study populations and settings. For example, the index study included students both in the early and later years of their training, whereas the study in South Africa included only first-year students. Being a first-year student may explain the significant knowledge gaps regarding CC. Contrary to earlier studies in Africa, in which mass media were the main source of information, health centers were cited as the primary information source [24,25].

In this study, most respondents (51.7%) didn’t know that HPV, a sexually transmitted virus, is the causative agent of CC. High institution scholars are unnecessarily assumed to be knowledgeable because of their continual exposure to various sources of information, including social media and the internet. The present study highlighted that 60.7% of participants were not aware of the national vaccination program against CC. Despite being learners in high learning institutions, nearly half of the participants (48.3%) did not know any CC screening method. In line with previous studies [8–11,13,14,18–27], the findings in our study suggest an urgent call for advocating and promoting health education, even among University scholars.

Access to the right information regarding CC empowers women to effectively fight against CC in support of the WHO’s strategy for CC eradication, which entails a target of a global coverage of 90% HPV vaccination of all girls and adolescents, 70% screening of women at risk, and 90% treatment of precancerous and invasive cancer by 2030 [28]. To achieve the elimination of CC in Africa, there is a need to increase access to education and primary and secondary preventive measure services for all women at risk of developing this disease. In this study, 79.9% of participants were able to identify at least one risk factor for CC. This was higher than what was reported in the previous studies that were conducted in Ethiopia and South Africa, which reported the participants’ awareness rates of CC risk factors of 40.5% and 6%, respectively [18,24]. Practicing safe sex (avoiding risky sexual behavior) and HPV vaccination were the most identified preventive measures for CC. The findings compare well with previous studies [8,10,11,14,20,22–24]. Most of the participants (75.7%) displayed a bad perception of the seriousness of CC. Similarly, the majority (70.3%) did not think that screening helps in the prevention of CC. Nearly similar results have been reported by previous African studies [8–10,12,13,17–21]. The findings suggest that CC health education is still low among University students. If access to continuous health education programs for all women at risk of developing CC is not made a priority and part and parcel of the CC elimination strategy, surely this issue will continue to be an important barrier towards the timely CC elimination targets [24,25].

Interestingly, the vast majority of the female respondents (82.5%) perceived that they were not at risk of getting CC, and if given an opportunity to be screened, a considerable proportion were either not sure (14.7%) or not ready (6.2%). Similar misconceptions were also reported in previous studies [18,20]. This can be attributed to the fact that educated people tend to think that they are exempt from CC, which has been associated with low socioeconomic status and discriminatory risky sexual behaviors. Regular checkups for all people are the key to the early detection of precancerous lesions and, thus, improve treatment outcomes [12,25,27]. Fear of the screening procedure and results was among the primary reasons given for not getting screened. Studies in Asia, Africa, and North America reported almost similar findings [8,10–14,18–27]. The main barrier to screening in Yemen was lack of knowledge of CC, among others [29]. A Ugandan study found that the primary barriers to screening practice were shyness, the belief that it was unnecessary, and fear of the gynecological procedure [30]. Walz and his co-workers in Somalia reported that only 46% of healthcare professionals knew that an HPV vaccine could prevent CC, and 89% of healthcare professionals disagreed that HPV vaccines were available to their patients [31]. The findings from the index study and these previous studies suggest that there is a need for continuous investment in health education regarding the prevention and control of CC in the women communities at risk of developing this disease, both in developing countries and marginalized communities in developed countries. Similarly, policymakers and other stakeholders in health should place a higher priority on the equipment required to conduct both disease screenings, increasing access to the HPV vaccine, and comprehending patients’ potential reasons for refusing screening.

Strangely, the majority of study participants (82.5%) in this study didn’t perceive that they were at risk of developing CC. It is important to find potential solutions to change this poor attitude and practices, as well as to explain and comprehend their underlying causes. The findings imply that future studies that use qualitative approaches are essential to investigate the reasons for women’s risk perception of CC. Although Pap smear cytology screening has contributed to a significant reduction of CC burden in western countries, unfortunately, this technique is not routinely available or seems unfavorable in most African countries with a high CC burden [4,24]. Instead, visual inspection of the cervix with acetic acid (VIA) is the standard screening method, and it entails a gynecological examination [4,5,9]. As highlighted earlier, barriers to screening uptake among under-screened women include stigma, unawareness of screening options, education level, and fear of the gynecological examination, as it may be considered embarrassing, invasive, painful, or culturally inappropriate by some women [32]. Furthermore, direct and indirect economic costs, such as transportation costs, lost income, and waiting time, have been reported to have a negative effect on CC screening attendance [33,34]. In addition, not feeling in danger, a lack of symptoms, negligence, a lack of interest, and the unpleasantness of the test have been identified as factors that negatively influence the uptake of CC screening [29–36]. However, the younger age of the vast majority of the study participants in our study (over 90% were under 25 years old) may have contributed to this observation. Women, particularly those of reproductive age, including University students, should not be skeptical about CC screening services that are currently available mainly in major academic centers in most developing countries.

The present study has established that socio-demographic characteristics such as marital status and age do not significantly correlate with having good KAP about CC control. Nevertheless, as expected, being a medical University student and in ≥4^th^ year of training were significantly correlated with better awareness of CC as compared to non-medical University and <4 year of study groups (p < 0.000). Our study results are consistent with previous research done in Africa and Asia [8–11,13,14,32]. Regardless of the type of degree program, students in medical institutions have higher chances of being exposed to medical-related knowledge in their learning environments and course of training compared to non-medical students. Likewise, students in advanced years of training are likely to be in clinical rotations, thus becoming more familiar with CC as compared to those in their earlier years of training. Additionally, we observed that females were more likely than their male counterparts to have a positive attitude (p < 0.000). These results imply that raising CC awareness and knowledge affects female students’ propensity to submit to CC testing and exhibit associated behavior [37]. The results of this study have some implications for CC research as well as health promotion and education initiatives. University students should participate in health promotion and education initiatives that focus on giving them factual information about this disease, its risk factors, symptoms, and effects, as well as the value of routine screening. Students’ knowledge on screening for CC is likely to change their attitude and practices for better results.

### Study limitations

This study has some limitations. Social desirability bias was a potential issue that this study may have run into due to self-administering the questionnaires. The other limitation of the study is that, because it is cross-sectional, the temporal order of events cannot be established.

In addition, the study was conducted at three Universities in one region only (the Kilimanjaro region). Thus, the results of this study may not necessarily apply to students at Universities in other regions across the country. In addition, the pre-existing knowledge of the students was not taken into account in this study. Lastly, but not least, the odds ratio had rather wide confidence intervals, indicating uncertainty or variability in the estimated effect or outcome. The wide confidence intervals observed in our study may be due to the high data variability, measurement errors, or skewed data.

## Conclusions

Most of the University students in the Kilimanjaro region of Tanzania lack a basic understanding of cervical cancer and its preventive measures. Thus, this gap needs to be filled in order to reduce the impact of this disease. The findings suggest the value of introducing health education initiatives, particularly among non-medical University students, so as to raise awareness and familiarity with cervical cancer. In order to develop healthy behavior and lower the incidence of this disease, health education interventions are constantly required to improve young women’s understanding of factors that increase the risk of cervical cancer and prevention methods before they engage in risky behaviors. Governments and non-governmental organizations should cooperate to increase University students’ awareness of cervical cancer screening in Tanzania. In order to accelerate cervical cancer elimination targets, no woman should be left behind.
